# Validation of the Portuguese version of the Evidence-Based Practice Questionnaire

**DOI:** 10.1590/0104-1169.0367.2561

**Published:** 2015

**Authors:** Rui Pedro Gomes Pereira, Ana Cristina Pinheiro Guerra, Maria José da Silva Peixoto de Oliveira Cardoso, Alzira Teresa Vieira Martins Ferreira dos Santos, Maria do Céu Aguiar Barbieri de Figueiredo, António Cândido Vaz Carneiro

**Affiliations:** 1 Doctoral student, Instituto de Ciências Biomédicas Abel Salazar, Universidade do Porto, Porto, Portugal. Adjunct Professor, Escola Superior de Enfermagem, Universidade do Minho, Braga, Portugal; 2 Doctoral student, Instituto de Ciências Biomédicas Abel Salazar, Universidade do Porto, Porto, Portugal. RN, Unidade de Cuidados Intensivos Polivalente, Hospital de Santo António, Centro Hospitalar do Porto, Porto, Portugal; 3 PhD, Associate Professor, Escola Superior de Enfermagem do Porto, Porto, Portugal; 4 PhD, Director, Centro de Estudos de Medicina Baseada na Evidência, Faculdade de Medicina, Universidade de Lisboa, Lisboa, Portugal

**Keywords:** Evidence-Based Nursing, Methods, Evidence-Based Practice

## Abstract

**OBJECTIVES:**

to describe the process of translation and linguistic and cultural validation of the Evidence Based Practice Questionnaire for the Portuguese context: Questionário de Eficácia Clínica e Prática Baseada em Evidências (QECPBE).

**METHOD:**

a methodological and cross-sectional study was developed. The translation and back translation was performed according to traditional standards. Principal Components Analysis with orthogonal rotation according to the Varimax method was used to verify the QECPBE's psychometric characteristics, followed by confirmatory factor analysis. Internal consistency was determined by Cronbach's alpha. Data were collected between December 2013 and February 2014.

**RESULTS:**

358 nurses delivering care in a hospital facility in North of Portugal participated in the study. QECPBE contains 20 items and three subscales: Practice (α=0.74); Attitudes (α=0.75); Knowledge/Skills and Competencies (α=0.95), presenting an overall internal consistency of α=0.74. The tested model explained 55.86% of the variance and presented good fit: χ2(167)=520.009; p = 0.0001; χ2df=3.114; CFI=0.908; GFI=0.865; PCFI=0.798; PGFI=0.678; RMSEA=0.077 (CI90%=0.07-0.08).

**CONCLUSION:**

confirmatory factor analysis revealed the questionnaire is valid and appropriate to be used in the studied context.

## Introduction

Evidence-based practice is defined as a process in which nurses make clinical decisions using the best scientific evidence available, their clinical experience and patients' preferences in the context of resources available^(^ 1 ^)^. A large systematic review conducted in 2004^(^ 2 ^)^ identified 630 papers published between 1972 and 2001, which addressed the use of evidence resulting from investigations regarding nursing practice. The conclusion was that, despite growing interest in elements that either hinder or facilitate the use of research, the field under study was relatively underdeveloped, justifying the development of additional conceptual work and support. Despite the expressive number of bibliometric findings identifying diverse studies^(^ 3 ^-^ 7 ^)^ on Evidence-Based Practice (EBP) and focusing on barriers, attitudes, practices, perceptions, and beliefs, among others, there is no broad set of instruments properly validated for the Portuguese context enabling rigorous and systematic assessment of the competencies of nurses concerning EPB and, consequently, enabling the structuring of interventions and implementation of strategies that favor its sustainable adoption in a more generalized manner. In this sense, multiple dimensions influence the processes of translating and incorporating evidence into clinical practice and these processes have been the focus of attention^(^ 8 ^)^ in the construction of assessment instruments. Specifically referring to the Evidence Based Practice Questionnaire, developed by Upton & Upton^(^ 9 ^)^ in 2006, information and opinions concerning the use of evidence-based practice were gathered from healthcare workers. Validating it to enable its generalized use is important since this instrument is currently recurrent in multiple contexts and there is, in addition to its original version in English, a Spanish version^(^ 10 ^)^ that was accomplished through a validation study conducted in 2009. Noting that its design and features denoted a high probability of the instrument being applicable in the nursing practice as developed in Portugal, this study was conducted to describe the process of translation and linguistic and cultural validation of the Evidence Based Practice Questionnaire for the Portuguese context, named *Questionário de Eficácia Clínica e Prática Baseada em Evidências* (QECPBE). It not only allows practices, attitudes, knowledge/abilities and competencies to be assessed, but also grounds interventions intended to improve proficiency in this field on the part of nursing workers.

## Method

The questionnaire's Portuguese version, *Questionário de Eficácia Clínica e Prática Baseada em Evidências*, is a self-administered instrument, the original version of which is comprised of 24 items scored through a semantic differential scale organized in three dimensions. The first component addressing Practices is scored on a Likert scale ranging from 1 (never) to 7 (frequently) and contains six items. Attitudes, the second component, is comprised of four items and the respondents score the items by choosing an answer that ranges between two opposite pairs of statements. Finally, the third component, designed to assess Knowledge/Skills and Competencies, is scored using a Likert scale, though answers range between 1 (worst) and 7 (best). The instrument's translation and adaptation included assessing its psychometric properties. After obtaining formal authorization from the authors of the original version, we proceeded to the translation of the questionnaire from English to Portuguese, which was performed by two independent translators. In this translation process, the semantic equivalence of some terms was verified. Afterwards, a panel of experts examined the conceptual equivalence of various items achieving consensus. The back translation was also performed by one independent translator and agreements and differences were verified. Finally, the instrument was analyzed in regard to its layout, appearance, legibility, and receptivity to content. 

A methodological cross-sectional study was conducted with an accidental sampling in a university hospital located in the North of Portugal. Considering the nature of the instrument, only nurses working full-time in clinical practice or those who, despite other activities, such as management, teaching or research, still worked most of time in clinical practice, were included. Data were collected in the following hospital departments or services: General Emergency, Intensive Care, Medicine, Surgery, Vascular Surgery, Pediatrics, Orthopedics, Urology, and Outpatient. The study project was approved and authorized by the Clinical Nursing Board, Institutional Review Board, and Board of Directors. A total of 995 self-administered questionnaires were distributed and 358 forms that were valid for the purposes of the study were returned. Hence, a response rate of 36% was obtained. The participants (n=358) voluntarily consented to participate in the study and the return of a valid and completed questionnaire was considered to constitute a participant's formal consent. Data were collected between December 2013 and March 2014.

The statistical analysis of data, i.e., parametric and multivariate analysis, was performed using SPSS version 22.0. The reliability of the subscales was assessed using Cronbach's alpha, a measure of internal consistency. Exploratory factor analysis was performed through Principal Component Analysis using orthogonal rotation according to the Varimax method. The verification of whether data were appropriate to this type of analysis was performed according to the Kaiser-Meyer-Olkin (KMO) criteria and Bartlet's test. The following criteria were utilized in the confirmation of the number of factors^(^ 11 ^)^: (1) eigenvalues >1; (2) exclusion of factor loads <0.40; (3) each factor should explain at least 5% of the variance; (4) application of the principle of discontinuity. Factor validity was assessed using Confirmatory Factor Analysis (CFA) with AMOS resources (version 21, SPSS-IBM). The existence of outliers was assessed by Mahalanobis squared distance and normality was assessed with an asymmetry coefficient and univariate and multivariate kurtosis. We considered as input the covariance matrix adopting the ML (Maximum Likelihood) method of estimation. The model's goodness of fit was evaluated according to the indexes and respective reference values^(^ 12 ^-^ 13 ^)^. Local goodness of fit was assessed using factor loads and the individual reliability of items. Goodness-of-fit index (GFI), Adjusted goodness-of-fit index (AGFI), Comparative Fit Index (CFI) and Root Mean Square Error Approximation (RMSEA) were used. The GFI, AGFI and CFI should be close to 0.90, while the recommended RMSEA is up to 0.08^(^ 12 ^-^ 13 ^)^. Model fitting to the theoretical considerations went beyond the modification indices. 

## Results

Most participants (n=358) were female (78%), aged between 30 and 39 years old (48.0%), and 49% had earned a bachelor's degree in nursing less than four years ago (year of graduation ≥ 2011) (Table 1). The instrument is composed of 24 items and admits only one out of seven possible responses. The number of participants was intended to fully meet the requirements concerning sampling size, as well as power and reliability criteria^(14)^

**Table 1 -:** Characterization of the sample according to sex, age, and time since graduation, Porto, Portugal, 2014

			n	%
Sex				
	Male		79	22.0
	Female		279	78.0
	Total		358	100
Age group				
	20-29		79	22
	30-39		172	48
	40-49		75	21
	50-59		32	9
	Total		358	100
	Year of graduation			
		≤ 2000	126	35
		2001 – 2010	57	16
		≥ 2011	175	49
		Total	358	100

The instrument's original version^(^ 9 ^)^ contains 24 items and three subscales: Practices (α=0.85); Attitudes (α=0.79); Knowledge/Skills and Competencies (α=0.91); it has an overall internal consistency of α=0.87. The principal component analysis suggested five dimensions that would explain 65.78% of the total variance, while Cronbach's was 0.84. Working with the three dimensions, however, in accordance with what is proposed by the authors of the original questionnaire and rejecting one item (P7) because it presents abnormal behavior overlapping components 1 and 2, we obtained a final Cronbach's α=0.74, which in this case explains 55.86% of the total variance. In this refinement process, we obtained the following Cronbach's alphas for each of the dimensions under study: Practices (α=0.74); Attitudes (α=0.75); Knowledge/Skills and Competencies (α=0.95). Table 2 presents the analysis of principal components in the version obtained with three dimensions. Note that the three dimensions presented here are equivalent to those proposed by the authors of the original study and are composed by the same items, with the exception of the one item excluded (P7 - My workload is too great for me to keep up to date with all the new evidence/ New evidence is so important that I make the time in my work schedule.)

**Table 2 -:** Principal components analysis (3 dimensions)

Item	Components		
	1	2	3
6. Partilhou essa informação com colegas	-.003	.580	-.036
5. Avaliou os resultados da sua prática	.122	.652	.039
4. Integrou as evidências que encontrou na sua prática	-.002	.692	.043
3. Analisou criticamente e segundo critérios explícitos, qualquer literatura que tenha encontrado	.019	.668	.017
2. Localizou as evidências relevantes após ter formulado a pergunta	.007	.718	.044
1. Formulou uma pergunta de partida claramente definida, como início de um processo para preencher essa lacuna	.018	.642	.025
11. Competências de pesquisa	.799	.031	-.027
12. Competências em TI (Tecnologias de Informação)	.700	.042	.002
13. Monitorização e revisão de competências práticas	.798	-.016	-.074
14. Conversão das suas necessidades de informação numa pergunta de investigação	.729	-.092	-.065
15. Percepção dos principais tipos e fontes de informação	.834	.038	-.029
16. Capacidade de identificar lacunas na sua prática profissional	.732	.067	.049
17. Saber como obter as evidências	.816	.004	.011
18. Capacidade de analisar, de forma crítica, as evidências segundo normas definidas	.865	.026	.011
19. Capacidade de determinar a validade (aproximação da verdade) do material	.831	-.022	-.021
20. Capacidade de determinar a utilidade (aplicabilidade clínica) do material	.843	.037	.029
21. Capacidade de aplicar a informação a casos individuais	.835	.043	.010
22. Partilha de ideias e informação com colegas	.725	.088	.147
23. Divulgação de novas ideias sobre os cuidados aos colegas	.703	.078	.110
24. Capacidade de rever sua própria prática	.744	.054	.094
8. Não me agrada que a minha prática clínica seja questionada / Acolho com agrado as perguntas sobre a minha prática	.051	-.031	.770
9. A prática com base em evidências é uma perda de tempo / A prática baseada em evidências é essencial à prática profissional	-.051	.028	.853
10. Mantenho-me fiel a métodos testados e aprovados, ao invés de mudar para algo novo / A minha prática mudou devido às evidências que encontrei	.079	.121	.815

The model suggested by the Exploratory Factor Analysis (EFA), which included three latent variables and 23 observable variables, was tested by CFA and showed poor fit. After reading the modification indices, a new model was devised in which some items were excluded (P22 - Sharing of ideas and information with colleagues; P23 - Dissemination of new ideas about care to colleagues; and P24 - Ability to review your ow*n practice*) was tested and goodness of fit was obtained: χ^2^ (167) = 520.009; p = 0.0001; χ^2^df = 3.114; CFI = 0.908; GFI = 0.865; PCFI = 0.798; PGFI = 0.678; RMSEA = 0.077 (CI 90%=0.07-0.08). All the factor loadings between latent and observed variables were statistically significant.

Table 3 presents the results of the confirmatory factor analysis of QECPBE-20's three-factor structure. It shows the items assigned to each of the dimensions upon which the Portuguese version of the instrument was based.

**Table 3 -:** -QECPBE-20's Confirmatory three-factor model

	Components		
	Conhecimento/Habilidades, Competências	Práticas	Atitudes
P6		.578	
P5		.653	
P4		.693	
P3		.670	
P2		.718	
P1		.643	
P11	.817		
P12	.723		
P13	.805		
P14	.762		
P15	.853		
P16	.702		
P17	.835		
P18	.871		
P19	.849		
P20	.850		
P21	.823		
P8			.776
P9			.855
P10			.822

**Figure 1 -:**
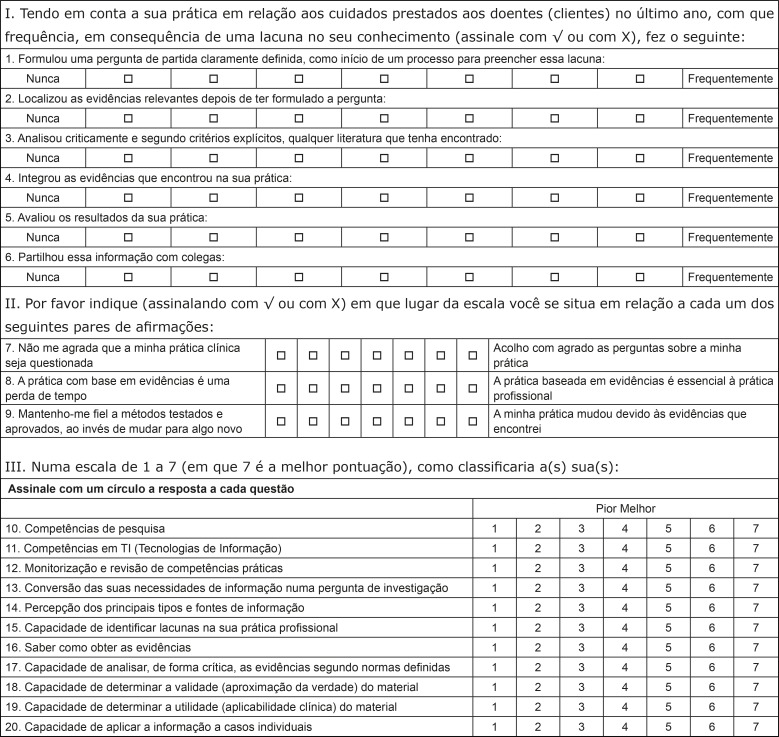
Questionário sobre Eficácia Clínica e Práctica Baseada em Evidências

Given the various analyses performed, Figure 1 presents the instrument's Portuguese version, QECPBE-20, composed by the subscales previously identified, including the initial explanatory framework concerning its use and self-administration. 

This questionnaire was conceived to collect information and opinions held by healthcare workers concerning the use of evidence-based evidence. There are no right or wrong answers, only interest in the participants' opinions and use of evidence in their practices.

## Discussion

According to the results, the QECPBE-20's three-factor model presents empirical evidence for its use in regard to construct validity, as well as in regard to reliability analysis of latent variables. Comparing the analysis of the Portuguese version with the original questionnaire^(^ 9 ^)^ and the Spanish version^(^ 10 ^)^, we verified general overlapping of results, while the Portuguese version obtained a final version with 20 items and statistical significance greater than that found for the Spanish version.

QECPBE-20 presented some limitations, if compared to other studies^(^ 3 ^-^ 4 ^,^ 8 ^,^ 15 ^-^ 16 ^)^ addressing instruments and the assessment of evidence-based practice, in regard to the dimensions included, particularly in regard to knowledge concerning clinical practice, change of evidence-based practice, and elements that facilitate change and skills. Similarly, the barriers against EBP are ignored in this instrument, even though significant importance is given to the incorporation of effective evidence-based nursing practice^(^ 6 ^)^, due to personal, professional, academic or organizational factors. Hence, the use of QECPBE-20 should be complemented by other instruments that are validated and available for the Portuguese context^(^ 15 ^,^ 17 ^)^. The joint application of instruments will enable the assessment of methodological competencies regarding EBP and allow its use in other spheres, related to education at this level and to the implementation of programs encouraging the integration of evidence with the delivery of care. On the other hand, these instruments can help outline the profile of workers required to make decisions^(^ 18 ^)^, while these workers should always ground their practice on the best scientific knowledge available. In this regard, and as already shown^(^ 18 ^-^ 19 ^)^, in order to perform safely and professionally, nurses require more knowledge, improved skills, and should be effectively confident when making decisions. As nurses gain confidence in their practice, they tend to know better how to incorporate research knowledge into practice.

Another aspect that should be further considered is related to the potential limitation brought by the context of the professional practice of the nurses addressed in this study; even though it is very significant and part of an academic context, is centered on a single hospital facility. Hence, further studies are needed, conducted in other contexts, such as primary healthcare, to verify whether the results are in agreement or not, as there are differences in terms of EBP from an organizational perspective.

## Conclusion

The analysis showed empirical evidence regarding the questionnaire and it is valid and appropriate to be used in the Portuguese context, with strong internal consistency. Considering the results, QECPBE-20 can be systematically disseminated and used.

The satisfactory results obtained in the validation process reinforce QECPVE-20's importance and practical implications. These implications are verified at various levels, as well as in education, such as promoting competencies and skills, and also in the direct delivery of care or in nursing research involving workers. The assessment of practices, attitudes, knowledge/skills and competencies should be a component of structural support and ground the definition of personalized interventions directed to groups and specific organizational contexts, aiming to promote and implement EBP among nurses.
